# Beyond apoptosis: harnessing natural products to target alternative regulated cell death for overcoming multidrug resistance in cancer

**DOI:** 10.3389/fonc.2026.1917061

**Published:** 2026-07-22

**Authors:** Chao Yang, Jia Wang, Baoyi Ni, Wei Zhan

**Affiliations:** 1 Hongqi Hospital Affiliated to Mudanjiang Medical University, Mudanjiang, China; 2 Mudanjiang Medical University, Mudanjiang, China

**Keywords:** multidrug resistance, nanomedicine, natural products, non-apoptotic regulated cell death, precision oncology

## Abstract

Acquired multidrug resistance (MDR) and apoptosis evasion severely limit the efficacy of conventional cancer therapies. Triggering non-apoptotic regulated cell death (RCD)—specifically ferroptosis, pyroptosis, cuproptosis, and disulfidptosis—offers a promising therapeutic paradigm to bypass these apoptotic blockades. Natural products (NPs) have emerged as potent modulators of these alternative RCD pathways owing to their unique structural diversity and multi-target network pharmacology. This review systematically delineates the molecular mechanisms by which phytochemicals disrupt cellular redox homeostasis and exploit metabolic vulnerabilities to execute non-apoptotic cytolysis. Furthermore, we highlight how NP-induced RCD triggers immunogenic cell death (ICD) and remodels the immunosuppressive microenvironment, thereby establishing a reciprocal reinforcement loop with host antitumor immunity. Despite compelling preclinical evidence, the clinical translation of NPs is hampered by pharmacokinetic limitations, target ambiguity, and adaptive resistance. To address these translational bottlenecks, we discuss the integration of smart nano-co-delivery platforms, artificial intelligence (AI)-driven structural optimization, and PROTAC technology. Ultimately, we emphasize the critical necessity of transitioning toward precision oncology frameworks through biomarker-guided patient stratification and pathway-specific pharmacodynamic monitoring, providing a comprehensive roadmap for leveraging NPs to combat recalcitrant malignancies.

## 1 Introduction

Conventional anticancer therapeutic modalities, including standard cytotoxic chemotherapy and molecularly targeted agents, primarily rely on the activation of programmed cell death pathways—specifically canonical apoptosis—to eradicate malignant populations (1). However, despite the initial clinical efficacy of these agents, malignant cells frequently undergo genetic and epigenetic alterations that result in acquired MDR, presenting a formidable impediment to successful oncology outcomes (2). A primary driver of this therapeutic failure is apoptosis resistance, whereby tumor cells establish redundant molecular survival networks to evade caspase-mediated death signals. Malignant cells achieve this resistance through several characterized mechanisms, most notably the transcriptional upregulation of anti-apoptotic Bcl-2 family proteins (e.g., Bcl-2, Bcl-xL, Mcl-1), loss-of-function mutations in the TP53 tumor suppressor gene, and the overexpression of Inhibitor of Apoptosis Proteins (IAPs) (2, 3). Because these molecular aberrations form highly interconnected and compensatory networks, single-agent pro-apoptotic induction is frequently rendered insufficient, necessitating the development of alternative therapeutic paradigms that bypass the established Bcl-2/IAP/p53 resistance axis (1, 3).

To address the limitations imposed by apoptosis resistance, recent oncology research has identified non-apoptotic regulated cell death pathways as promising alternative strategies (4). These alternative RCD pathways—specifically ferroptosis, pyroptosis, cuproptosis, and disulfidptosis—operate via metabolic, biochemical, and morphological mechanisms that are fundamentally distinct from traditional apoptosis (5). Crucially, because these alternative RCD pathways operate through biochemical vulnerabilities that do not rely on the classical mitochondrial outer membrane permeabilization (MOMP) or canonical caspase cascades, they remain fully operational in cells harboring mutations that induce apoptosis resistance (4, 5).

The specific upstream molecular cascades governing these alternative RCD pathways underscore their distinct therapeutic utilities. Ferroptosis is an iron-dependent form of cell death driven by the unrestricted accumulation of lethal lipid peroxides (6). It is tightly controlled by the cystine/glutamate antiporter (system X^c-^) and glutathione peroxidase 4 (GPX4); specifically, inhibition of GPX4 leads to the lethal accumulation of unchecked lipid peroxides, whereas inhibition of SLC7A11 (the core component of system X^c-^) directly blocks glutathione (GSH) synthesis, both of which independently culminate in ferroptotic cell death (6). Pyroptosis represents a highly inflammatory form of lytic programmed cell death mediated by the gasdermin protein family (7). Whether triggered by canonical inflammasome activation or the conversion of apoptotic stimuli via Caspase-3, the proteolytic cleavage of Gasdermin D (GSDMD) or Gasdermin E (GSDME) releases an N-terminal domain that oligomerizes within the plasma membrane to form lethal pores (7, 8). Cuproptosis involves an intracellular copper-dependent toxic mechanism where excess copper ions bind directly to lipoylated components of the mitochondrial tricarboxylic acid (TCA) cycle, specifically dihydrolipoamide acetyltransferase (DLAT) (9). Modulated by the upstream reductase ferredoxin 1 (FDX1), excessive copper induces the aggregation of these lipoylated proteins, causing severe proteotoxic stress and rapid mitochondrial dysfunction (9, 10). Finally, disulfidptosis is a recently identified metabolic cell death modality triggered under glucose-starved conditions in cells overexpressing SLC7A11 (11). Glucose deficiency depletes intracellular NADPH, leading to the accumulation of unreduced disulfides. This induces abnormal intermolecular disulfide cross-linking among actin cytoskeleton proteins, resulting in actin filament collapse and rapid cell death (11).

Beyond direct tumor cell cytotoxicity, the induction of specific alternative RCD pathways modulates the tumor immune microenvironment through the mechanism of ICD (12). Unlike classical apoptosis, which is immunologically silent, the lytic nature of pyroptosis and the membrane disruption characteristic of ferroptosis induce the active or passive release of damage-associated molecular patterns (DAMPs), including high-mobility group box 1 (HMGB1), adenosine triphosphate (ATP), and cell-surface calreticulin (12, 13). These chemotactic signals recruit and promote the maturation of dendritic cells (DCs), which subsequently stimulate the clonal expansion and infiltration of CD8+ cytotoxic T lymphocytes (CTLs) into the tumor parenchyma. By altering the local cytokine profile, alternative RCDs shift the microenvironment from an immunosuppressive to an immunoreactive state, thereby providing a mechanistic rationale for combined immunotherapeutic regimens (12, 13).

In the search for effective modulators capable of triggering these non-apoptotic RCD pathways, NPs and components derived from traditional Chinese medicine (TCM) have emerged as highly viable candidates. Historically, natural scaffolds have served as a foundation for cancer pharmacology, contributing to approximately 80% of FDA-approved anticancer drugs over the past three decades (14). NPs offer unique pharmacological advantages, including remarkable structural diversity, multi-target polypharmacology, and favorable systemic safety profiles (15). Unlike synthetic single-target inhibitors, which are susceptible to compensatory bypass mutations, NPs often demonstrate network pharmacology. They can simultaneously engage multiple signaling nodes, disrupting redundant survival pathways and reducing the probability of acquired resistance (14, 15).

Accumulating preclinical evidence indicates that various phytochemical classes can act as epigenetic or transcriptional modulators to downregulate SLC7A11, activate Caspase-3/GSDME axes, or alter copper homeostasis. For example, representative natural compounds identified in recent literature—such as shikonin (activating the ROS/BAX/caspase-3/GSDME pyroptotic axis), dihydroartemisinin (DHA) (inhibiting System X^c-^ and depleting NADPH), curcumin, and quercetin—have demonstrated potent efficacy in triggering non-apoptotic pathways across various therapy-resistant cancer models (16, 17). When administered alongside conventional targeted therapies or platinum-based agents, these NPs function as potent chemosensitizers while reversing established multidrug resistance. However, the clinical translation of raw NPs is frequently restricted by pharmacokinetic limitations. To resolve these barriers, advanced nanodelivery platforms—such as self-assembled copper chlorogenic acid nanoparticles and metal-free cascade nanoreactors—have been integrated to enhance systemic stability and ensure precise tumor-targeted delivery (18, 19).

### 1.1 Literature search strategy, scoping criteria, and conceptual novelty

To ensure a rigorous and objective synthesis of the literature, a structured search strategy was deployed across PubMed and Web of Science databases for records published up to 2026. Search equations utilized Boolean operators linking terms such as “natural products”, “phytochemicals”, “multidrug resistance”, “MDR”, “ferroptosis”, “pyroptosis”, “cuproptosis”, and “disulfidptosis”. Studies were prioritized based on stringent inclusion criteria requiring direct experimental validation of alternative RCD execution (e.g., genetic knockdown/overexpression or specific pharmacological rescue assays), whereas studies relying solely on descriptive, indirect, or generic oxidative stress markers were excluded.

Among the expanding spectrum of non-apoptotic death programs, ferroptosis, pyroptosis, cuproptosis, and disulfidptosis were deliberately selected as the core mechanistic framework of this review. Unlike necroptosis or autophagy-dependent cell death—whose tumor-suppressive roles have already been extensively summarized in standalone literatures—these four emerging pathways represent the latest paradigm shifts in metabolic, redox, and ion-homeostasis-driven cytolysis. Crucially, they operate via distinct biochemical vulnerabilities that are completely uncoupled from the canonical Bcl-2/IAP/p53 apoptosis axis, making them uniquely suited for targeting apoptosis-resistant MDR phenotypes. Other modalities, such as necroptosis, are discussed complementarily within the context of multi-drug chemosensitization crosstalk rather than as primary thematic pillars. Distinct from conventional descriptive aggregations of phytochemical data, the specific conceptual advance of this review lies in its pioneering three-dimensional integration: systematically mapping the multi-target network pharmacology of natural scaffolds onto the execution machinery of these four emerging RCDs, and translating these mechanistic intersections into advanced nanomedicine co-delivery platforms and biomarker-guided precision oncology frameworks to provide a definitive roadmap for overcoming drug resistance.

## 2 Mechanisms of alternative regulated cell death pathways in cancers

**Figure 1:**
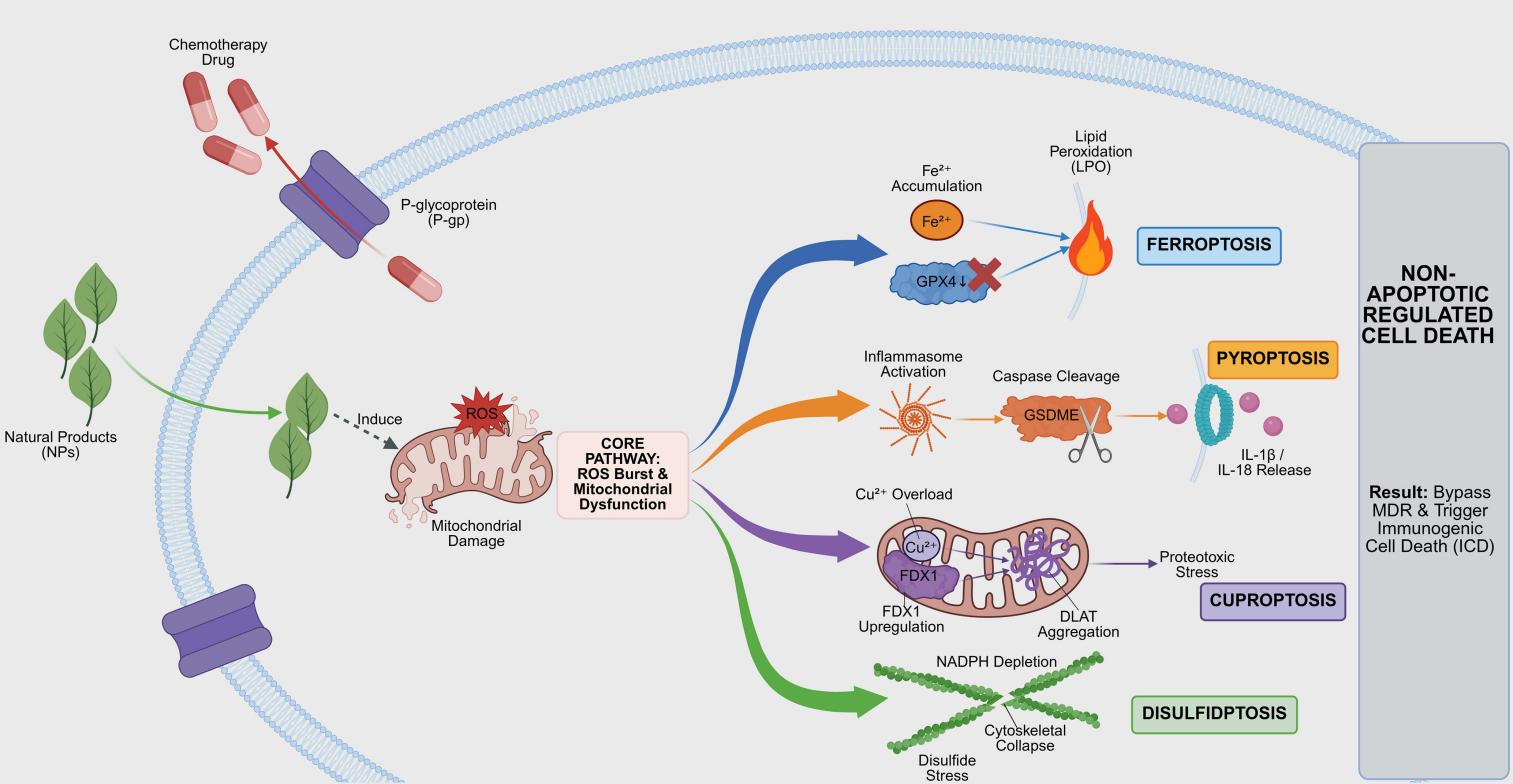
Molecular mechanisms of non-apoptotic RCD pathways modulated by natural products in cancer cells (created with BioRender.com). The schematic illustrates four distinct alternative RCD modalities: (1) Ferroptosis, driven by iron-dependent lipid peroxidation (PUFA-PL-OOH) and the inhibition of the SLC7A11/GSH/GPX4 antioxidant axis; (2) Pyroptosis, characterized by Caspase-mediated Gasdermin (GSDMD/GSDME) cleavage and subsequent membrane pore formation; (3) Cuproptosis, triggered by intracellular copper accumulation and FDX1-mediated aggregation of lipoylated DLAT within the TCA cycle; and (4) Disulfidptosis, executed via actin cytoskeleton collapse induced by severe disulfide stress under glucose deprivation. Natural products (NPs) function as multi-target modulators to bypass apoptosis resistance by specifically disrupting these critical metabolic and redox nodes.

As established above, non-apoptotic programmed cell death pathways present alternative mechanistic frameworks to eliminate therapy-resistant malignancies. The upstream regulatory cascades and downstream effector events of these four distinct pathways are comprehensively delineated below (**Figure 1**).

However, it is critical to acknowledge that the assignment of specific RCD modalities in preclinical studies often relies on partial or correlative markers. Throughout this review, rigorous evidence criteria are emphasized for validating RCDs: canonical ferroptosis must demonstrate lethal lipid peroxidation that is reversible by specific lipophilic antioxidants (e.g., ferrostatin-1 or liproxstatin-1) or iron chelators; pyroptosis requires biochemically confirmed gasdermin cleavage combined with pore-forming membrane rupture; cuproptosis necessitates copper dependence accompanied by FDX1-mediated DLAT lipoylation and subsequent protein aggregation; and disulfidptosis strictly requires the triad of glucose deprivation, high SLC7A11 expression, and catastrophic actin-cytoskeleton disulfide cross-linking. Studies lacking these definitive rescue or genetic ablation validations should be interpreted with caution.

### 2.1 Ferroptosis

Ferroptosis is an iron-dependent form of regulated cell death driven by the lethal accumulation of lipid peroxides on cellular membranes (20, 21). Iron metabolism dysregulation serves as the primary driver for this process. Malignant cells frequently upregulate transferrin receptor 1 (TFRC) to maximize iron uptake, while nuclear receptor coactivator 4 (NCOA4)-mediated ferritinophagy selectively degrades ferritin to release massive quantities of stored iron, thereby exponentially expanding the intracellular labile iron pool (LIP) (21, 22). Excess Fe^2+^ actively participates in Fenton chemistry, generating highly ROS that propagate catastrophic lipid peroxidation cascades (23). Regarding the generation of lethal lipid substrates, acyl-CoA synthetase long-chain family member 4 (ACSL4) catalyzes the activation of polyunsaturated fatty acids (PUFAs), which are subsequently esterified into membrane phospholipids by lysophosphatidylcholine acyltransferase 3 (LPCAT3) (24, 25). These formed PUFA-phospholipids undergo enzymatic oxidation by lipoxygenases (ALOXs) to generate toxic lipid hydroperoxides (24). To counteract this lipid peroxidation, tumor cells primarily rely on the system X^c-^/GSH/GPX4 antioxidant axis (6). System X^c-^ (consisting of the SLC7A11 subunit) imports extracellular cystine for GSH biosynthesis (21). GPX4 then utilizes GSH as an essential cofactor to reduce lethal lipid hydroperoxides into non-toxic lipid alcohols (6, 21). Furthermore, in therapy-resistant cancers, GPX4-independent defense mechanisms are frequently hyperactivated, including the FSP1-CoQ10 pathway and the DHODH-mediated mitochondrial defense, which coordinately orchestrate resistance to ferroptotic execution (26, 27).

### 2.2 Pyroptosis

Pyroptosis represents a highly inflammatory, lytic form of programmed cell death mediated by the precise proteolytic cleavage of the GSDM protein family (28, 29). The canonical pyroptotic signaling cascade is initiated by cytoplasmic pattern recognition receptors (e.g., NLRP3, AIM2), which recruit the ASC adaptor to assemble the inflammasome complex and subsequently activate Caspase-1 (28, 30). In contrast, the non-canonical pathway is triggered by the direct sensing of cytosolic lipopolysaccharide (LPS) by Caspase-4/5/11, prompting potassium efflux that secondarily activates the NLRP3 inflammasome (30). Both cascades converge on the cleavage of GSDMD, liberating its cytotoxic N-terminal domain (GSDMD-NT) (31). Following critical S-palmitoylation modifications, GSDMD-NT translocates to the plasma membrane, binds to acidic phospholipids, and undergoes extensive oligomerization to form transmembrane pores (10–18 nm in diameter) (32, 33). Crucially, terminal plasma membrane rupture (PMR) during pyroptosis is actively orchestrated by the trans-membrane protein ninjurin-1 (NINJ1), which undergoes filamentous oligomerization to drive the final catastrophic tearing of the membrane (34, 35). Within the tumor microenvironment, GSDME serves as another critical pyroptotic executor (36). GSDME is specifically cleaved by apoptosis-associated Caspase-3 or lymphocyte-derived Granzyme B, fundamentally redirecting an immunologically silent apoptotic response into highly immunogenic pyroptosis, culminating in the robust release of DAMPs (36, 37).

### 2.3 Cuproptosis

Cuproptosis is a mitochondria-dependent cell death modality triggered by the direct toxic interaction between excess intracellular copper ions and lipoylated metabolic enzymes within the TCA cycle (38). Upon cellular entry via the high-affinity copper transporter SLC31A1 (CTR1) or through synthetic exogenous copper ionophores, intracellular cupric ions Cu^2+^ are rapidly reduced to the highly reactive cuprous state Cu^+^ by FDX1 (39, 40). FDX1 functions as the master upstream regulator, exerting a dual physiological role: it mediates copper reduction while simultaneously facilitating protein lipoylation by promoting the activity of lipoyl synthase (LIAS) (41, 42). The newly generated Cu^+^ directly and specifically binds to the dithiolane rings of lipoylated DLAT, a core component of the pyruvate dehydrogenase complex (42, 43). This aberrant metal-protein binding induces severe disulfide bond-dependent oligomerization and the insoluble aggregation of these critical metabolic enzymes (38, 43). The aggregation of DLAT abolishes pyruvate dehydrogenase complex activity, resulting in the immediate collapse of the TCA cycle and generating overwhelming proteotoxic stress (41). Concurrently, the excessive accumulation of copper ions actively destabilizes vital iron-sulfur (Fe-S) cluster proteins, thereby paralyzing the mitochondrial electron transport chain and triggering rapid metabolic arrest (38, 44).

### 2.4 Disulfidptosis

Disulfidptosis is an inherently metabolic cell death modality particularly prevalent in tumor cells exhibiting high baseline expression of the cystine/glutamate antiporter SLC7A11, specifically executed via extreme disulfide stress-induced actin cytoskeleton collapse under conditions of glucose starvation (45, 46). In the context of cancer metabolism, SLC7A11 presents a lethal paradox (47). Under nutrient-replete conditions, high SLC7A11 expression robustly imports cystine to synthesize glutathione, effectively suppressing ferroptosis (46). However, when glucose supply is restricted, the pentose phosphate pathway (PPP) is abruptly arrested, causing a severe depletion of the intracellular NADPH pool (48). NADPH is the indispensable reducing equivalent required to convert imported cystine into soluble cysteine (45, 48). Consequently, the sustained importation of extracellular cystine driven by overexpressed SLC7A11 leads to the massive accumulation of unreduced intracellular disulfides (45). This profound disulfide stress completely overwhelms the cellular redox buffering capacity, inciting aberrant intermolecular disulfide cross-linking among critical structural components of the actin cytoskeleton, most notably targeting the highly reactive Cys374 residue of actin filaments (45, 49). Because the active polymerization of the F-actin network is continuously driven by the Rac1-activated WAVE regulatory complex (WRC) and the Arp2/3 axis, these dynamic cytoskeletal infrastructures are exceptionally vulnerable to covalent cross-linking (50). The sudden rigidification of the actin network triggers a catastrophic structural collapse, leading to immediate plasma membrane detachment and rapid lytic death (45, 50).

## 3 Natural products targeting alternative RCD pathways in cancers: pharmacological insights and paradigms

While NPs and bioactive components derived from TCM exhibit multi-target network pharmacology that can disrupt redundant survival signaling, claims of their “precise targeting” must be critically evaluated. The extensive polypharmacology of phytochemicals often results in correlative, rather than causative, marker alterations. Therefore, robust target engagement assays, coupled with genetic rescue (e.g., CRISPR-Cas9 knockout) or pharmacological ablation, are essential to distinguish direct, primary mechanism-of-action interactions from secondary downstream stress responses when evaluating NP-induced non-apoptotic RCD trajectories. By critically validating these complex molecular networks, these alternative pathways can be rationally leveraged to circumvent canonical apoptotic blockades in therapy-resistant malignancies. The representative phytochemicals, their specific molecular targets, and the corresponding non-apoptotic RCD pathways they trigger are comprehensively summarized in **Table 1**.

**Table 1:** Representative natural products targeting non-apoptotic RCD pathways in cancer therapeutics.

Phytochemical/NP	Target RCD pathway	Primary molecular target(s)/signaling axis	Specific pharmacological mechanism of action	Context/preclinical models	Ref.
DHA	Ferroptosis	Fe2+, GPX4, TFRC	Generates ROS via endoperoxide bridge Fenton chemistry; promotes autophagy-dependent GPX4 degradation and expands the labile iron pool.	Gastric cancer, triple-negative breast cancer (TNBC)	(51–55)
Resveratrol	Ferroptosis	ERK1/2–SGK1, NEDD4L	Suppresses the ERK1/2–SGK1 pathway to activate NEDD4L E3 ligase, directing GPX4 toward proteasomal degradation.	TNBC	(56)
Tanshinone IIA	Ferroptosis	p53, SLC7A11	Epigenetically upregulates p53, which directly binds to the SLC7A11 promoter to repress system Xc- transcription.	Gastric cancer	(57)
Curcumin	Ferroptosis/Cuproptosis	P62–KEAP1–NRF2, FDX1	Downregulates antioxidant targets (SLC7A11, GPX4) to induce lipid peroxidation; functions as an exogenous copper ionophore.	Non-small cell lung cancer (NSCLC), colorectal cancer	(58–61, 79)
Baicalein	Ferroptosis	JAK2/STAT3/GPX4	Blocks the JAK2/STAT3 cascade to suppress GPX4 transcription in a context-dependent manner (pro-ferroptotic in tumors).	Colorectal cancer, acute myeloid leukemia (AML)	(62, 63)
Shikonin	Pyroptosis	ROS, Caspase-3, GSDME	Induces BAX/Cytochrome c mitochondrial pathway to activate Caspase-3, which subsequently cleaves GSDME to execute lytic pyroptosis.	EGFR-mutant lung cancer	(70, 71)
Oridonin	Pyroptosis	Caspase-3, GSDME, NLRP3	Triggers Caspase-3/GSDME-mediated cytolysis in tumor cells while covalently inhibiting NLRP3 assembly in stromal immune cells.	Pancreatic ductal adenocarcinoma	(73, 74)
Ginsenoside Rh3	Pyroptosis/Ferroptosis	STAT3/p53/NRF2, Caspase-1	Exerts dual-lethality by exhausting GSH (ferroptosis) and canonically activating Caspase-1/GSDMD cleavage (pyroptosis).	Colorectal cancer	(77)
Emodin	Cuproptosis	FDX1, DLAT	Transcriptionally upregulates FDX1 and accelerates copper-dependent DLAT lipoylation and mitochondrial aggregation.	Hepatocellular carcinoma (HCC)	(78)
Celastrol	Cuproptosis	Copper coordination, GSH	Forms copper complexes for intracellular delivery while actively scavenging GSH, neutralizing endogenous copper-chelating defenses.	Various solid tumors (Nanomedicine applications)	(81)
Gaudichaudione H	Disulfidptosis	NRF2, SLC7A11	Forces paradoxical SLC7A11 overexpression under glucose starvation, exhausting NADPH and triggering actin cytoskeleton cross-linking.	HCC	(85)
Apigenin	Disulfidptosis	GLUT1	Acts as a competitive inhibitor of GLUT1 to enforce severe intracellular glucose deprivation, precipitating extreme disulfide stress.	Pancreatic cancer	(86, 87)

### 3.1 Exploiting redox vulnerabilities: multi-tiered induction of ferroptosis

Therapy−resistant cancer cells frequently exhibit elevated basal oxidative stress, rendering them highly dependent on robust antioxidant networks—foremost the system Xc^−^ (SLC7A11)/GSH/GPX4 axis (17). To systematically disrupt this multilayered antioxidant defense barrier, phytochemicals generally employ multi−target network pharmacology rather than single−target precision. However, to establish true pathway causality rather than mere correlative marker alterations, recent rigorous investigations mandate that NP−induced ferroptotic phenotypes must exhibit lethal lipid peroxidation that is actively rescued by specific lipophilic antioxidants (e.g., ferrostatin−1 or liproxstatin−1) or iron chelators. Validated phytochemicals disrupt ferroptotic defenses through two distinct pharmacological strategies: direct biochemical disruption/post−translational elimination of protective proteins, or upstream modulation of transcriptional/epigenetic landscapes.

#### 3.1.1 Direct chemical interaction and post−translational regulation – artemisinin derivatives

Artemisinin and its semi−synthetic derivative DHA exploit the iron−dependent nature of malignant cells. Rather than merely suppressing gene expression, DHA utilizes its endoperoxide bridge to react directly with intracellular ferrous iron (Fe^2+^), propagating a cascade of hydroxyl radicals (51). This chemical reaction is augmented by DHA-mediated upregulation of TFRC through the IRP/IRE regulatory axis and the induction of non−canonical, autophagy−independent ferritinophagy, which expands the labile iron pool (52, 53). Concurrently, DHA facilitates the downregulation of SLC7A11 and promotes the ubiquitin−mediated, autophagy−dependent degradation of GPX4 protein, triggering lethal lipid peroxidation (53, 54). This membrane damage is further enhanced by DHA−induced formation of the PEBP1/15−lipoxygenase (15−LO) complex, which selectively oxidizes phosphatidylethanolamine species (55). Through this dual mechanism—direct Fenton radical generation combined with targeted GPX4 ablation—DHA functions as a direct chemical perpetrator that operates independently of baseline transcriptional plasticity.

#### 3.1.2 Post−translational GPX4 ablation via ubiquitin−proteasome pathways

A distinct post−translational strategy is exemplified by resveratrol, which achieves GPX4 suppression without directly generating ROS. Resveratrol selectively downregulates the ERK1/2–SGK1 signaling pathway, thereby preventing the inactivation of the E3 ubiquitin ligase NEDD4L and directing GPX4 toward proteasomal degradation (56). This mechanism highlights how certain polyphenols can eliminate the core peroxidase at the protein−turnover level, contrasting with transcriptional repressors that act upstream of protein synthesis.

#### 3.1.3 Transcriptional reprogramming of system X^c−^ and antioxidant genes

A second major class of phytochemicals intervenes at the genomic level to curb cystine uptake and GSH biosynthesis. Tanshinone IIA functions as a transcriptional regulator by upregulating p53 expression, which binds directly to the promoter region of SLC7A11 to repress its transcription and reduce GSH biosynthesis (17, 57).

Similarly, curcumin inactivates the upstream P62–KEAP1–NRF2 signaling cascade, leading to coordinate downregulation of downstream antioxidant target genes including SLC7A11, GPX4, and HO−1 (58, 59).

However, this highlights the highly context−dependent nature of NP pharmacology. Depending on the specific cellular context and baseline redox thresholds, curcumin can exert the exact opposite effect in certain pulmonary adenocarcinoma models, inducing NRF2 nuclear translocation and upregulating heme oxygenase−1 (HMOX1) to promote massive heme degradation and free iron toxicity (60). Furthermore, curcumin modulates alternative intracellular pathways by activating JNK signaling and upregulating the glutamine transporter SLC1A5 to accelerate lipid ROS accumulation (61). These observations illustrate that transcriptional modifiers are heavily influenced by the tumor’s basal mutational and metabolic state.

#### 3.1.4 Flavonoids and alkaloids – convergent ferroptotic endpoints through divergent upstream nodes

Beyond the aforementioned scaffolds, specific flavonoids and alkaloids also converge on ferroptosis but engage distinct upstream regulators. Baicalein mediates context−dependent biological outcomes, functioning as an inhibitor of ferroptosis under physiological conditions to mitigate oxidative tissue injury, but acting as a pro−ferroptotic agent at elevated concentrations in malignant cells by blocking the JAK2/STAT3/GPX4 signaling cascade (62, 63). Luteolin induces autophagy−dependent ferroptosis by downregulating the iron exporter ferroportin (SLC40A1), trapping iron intracellularly and exacerbating Fenton chemistry (64). Quercetin downregulates system X^c−^ and GPX4 by repressing NRF2 while simultaneously activating the p−CaMK2/p−DRP1 axis to enhance mitochondrial ROS production (65). The isoquinoline alkaloid berberine suppresses the SLC7A11/SLC3A2/GPX4 axis in a p53−dependent manner and binds directly to Gli1 to inhibit Gli1/STAT3 signaling, revealing a Hedgehog−pathway intersection that is not observed with the aforementioned compounds (66–68).

Comparative pharmacology and context−dependent duality: A critical comparative evaluation of these modulators reveals distinct mechanistic divergence despite their convergence on ferroptotic endpoints. Artemisinin derivatives (e.g., DHA) fundamentally function as direct chemical perpetrators, leveraging their inherent Fenton−reactivity to propagate lipid peroxidation independently of baseline protein transcription. In contrast, polyphenols (e.g., resveratrol, curcumin) and flavonoids (e.g., baicalein, quercetin) function predominantly as transcriptional or epigenetic orchestrators. Consequently, the former class exerts rapid, stoichiometric oxidative stress, whereas the latter heavily relies on the basal transcriptional plasticity of the tumor cell, rendering them highly susceptible to the aforementioned context−dependent biphasic effects—acting as pro−oxidants in malignancies but preserving antioxidant capacity in healthy tissues. This dichotomy has direct translational implications: tumors with constitutively high NRF2 activity may respond more favorably to direct perpetrators like DHA, whereas those retaining p53−wild−type status might be preferentially sensitized by transcriptional repressors such as tanshinone IIA or berberine, although such hypotheses require dedicated validation in stratified preclinical models.

### 3.2 Reprogramming the apoptosis-to-pyroptosis switch for tumor immunogenicity

The induction of pyroptosis by natural products presents a distinct therapeutic advantage over classical apoptosis by converting an immunologically silent form of cell clearance into ICD, which promotes the release of DAMPs into the tumor microenvironment (69). Pharmacologically, natural compounds manipulate this death transition through two parallel programmatic axes: the direct amplification of canonical apoptotic cascades to trigger secondary GSDME cleavage, or context-dependent, cell-type-specific modulation of inflammasome platforms.

#### 3.2.1 Engagement of the Caspase-3/GSDME axis

In malignancies maintaining robust baseline expression of GSDME, specific phytochemicals exploit caspase-3 activation to execute the apoptosis-to-pyroptosis switch. Shikonin induces substantial intracellular ROS accumulation, which drives BAX mitochondrial translocation and subsequent cytochrome c release (70). This sequence initially activates the canonical mitochondrial apoptosis pathway; however, the resulting activation of the Caspase-9/Caspase-3 cascade does not culminate in silent apoptosis. Instead, the fully operational Caspase-3 specifically cleaves GSDME at the Asp270 residue to liberate its pore-forming N-terminal fragment (GSDME-N), hijacking the apoptotic machinery to execute cellular swelling and lytic membrane rupture (70, 71). This execution phase is modulated by concurrent cytoprotective autophagy via the ROS-dependent MAPK14/p38α pathway, and genetic or pharmacological inhibition of this autophagic flux significantly amplifies shikonin-induced pyroptosis (70). In tyrosine kinase inhibitor-resistant lung cancers, shikonin downregulates COX-2 via proteasomal degradation, suppressing PDK1/AKT and ERK1/2 pathways to activate Caspase-3-mediated GSDME cleavage (71). Similarly, DHA induces pyroptotic transitions in esophageal squamous cell carcinoma by downregulating pyruvate kinase M2 (PKM2), which activates Caspase-8 and Caspase-3 to drive GSDME-mediated pore formation (72).

#### 3.2.2 Context-dependent inflammasome modulation by oridonin

The kaurane diterpenoid oridonin demonstrates a highly differentiated, cell-type-specific pharmacological profile. Within stromal and immune cell compartments, oridonin acts as a specific covalent inhibitor of the NLRP3 inflammasome by binding to Cys279 in the NLRP3 NACHT domain, blocking the NLRP3–NEK7 macromolecular interaction required for inflammasome assembly (73). This suppresses systemic inflammation and downregulates tumoral PD-L1 expression via STAT3 inhibition to augment anti-tumor immunity (73). Conversely, within specific malignant cell lineages such as pancreatic ductal adenocarcinoma, oridonin and its structural analog ponicidin directly activate Caspase-3 to cleave endogenous GSDME, driving lytic membrane rupture and cytolysis (74). This capacity to suppress deleterious stromal inflammation while executing direct tumor cytolysis exemplifies the context-dependent utility of specific natural scaffolds.

#### 3.2.3 Canonical activation and polypharmacological crosstalk

Unlike the majority of GSDME-targeting agents, curcumin robustly engages the canonical pyroptotic infrastructure. In colorectal cancer models, curcumin activates Caspase-1 through the upregulation of NLRP3 inflammasome components, driving the proteolytic cleavage and membrane translocation of GSDMD (75). This Caspase-1 dependency is verified by the specific inhibitor VX-765, which blocks curcumin-mediated membrane swelling and lactate dehydrogenase release (75). In acute myeloid leukemia, curcumin upregulates the ISG3 transcription factor complex to activate NLRC4, AIM2, and IFI16 inflammasomes, executing GSDMD-dependent pyroptosis in a cell line-specific manner governed by baseline GSDMD expression (76). In addition to canonical signaling, specific saponins such as ginsenoside Rh3 engage in polypharmacological crosstalk by suppressing the STAT3/p53/NRF2 axis (77). This simultaneous exclusion of nuclear NRF2 downregulates HO-1 to activate NLRP3/Caspase-1/GSDMD-mediated pyroptosis, while concurrently downregulating SLC7A11 to deplete GSH and induce synchronized ferroptotic lipid peroxidation (77).

### 3.3 Sabotaging metabolic vulnerabilities: the frontiers of cuproptosis and disulfidptosis

Targeting cuproptosis and disulfidptosis represents an advanced strategy in natural product pharmacology, shifting the focus from canonical protein inhibition toward the exploitation of rigid metabolic constraints and structural vulnerabilities. Because these two non-apoptotic modalities are intrinsically tied to metabolic and energetic homeostatic imbalances, current pharmacological models have evolved beyond single-molecule inhibition toward direct disruption of mitochondrial regulatory complexes or metabolic interception leveraging nutrient-deprived states to induce structural infrastructure collapse.

#### 3.3.1 Mitochondrial complex disruption and exogenous ionophore mechanisms

The anthraquinone emodin induces cuproptosis in hepatocellular carcinoma by modulating the SLC7A11/FDX1 axis (78). Emodin upregulates FDX1 while concurrently downregulating SLC7A11 and GPX4, enhancing intracellular copper accumulation and promoting FDX1-mediated lipoylation and subsequent aggregation of DLAT within the mitochondrial matrix (78). This aggregation disrupts pyruvate dehydrogenase complex activity, causing TCA cycle failure and severe proteotoxic stress (78). Alternatively, natural polyphenols and triterpene structures can function directly as exogenous copper ionophores. Curcumin facilitates intracellular copper accumulation and upregulates components of the mitochondrial lipoylation pathway, bypassing endogenous antioxidant defenses under copper stress (79). Furthermore, a synthetic curcuminoid derivative, PBPD, enhances this effect by inhibiting the Notch1/RBP-J cascade to repress NRF2, which removes transcriptional inhibition on FDX1 and significantly amplifies copper-dependent DLAT aggregation (80). Similarly, celastrol exhibits strong coordination capacity with copper ions, functioning as a dual-action agent that delivers copper while simultaneously scavenging intracellular GSH to neutralize the primary endogenous copper-chelating defense (81). This self-amplified cuproptosis induces immunogenic cell death and activates anti-tumor immune responses (81). Other natural structures, including chagosendine C and pochinon D, induce mitochondrial dysfunction by targeting FDX1 or covalently binding to Cys173 of peroxiredoxin 1 (PRDX1) (82, 83). Additionally, quercetin binds directly to FDX1, working synergistically with exogenous copper complexes to restore cuproptosis sensitivity and reverse targeted therapy resistance in hepatic malignancies (84).

#### 3.3.2 Exploiting metabolic paradoxes for cytoskeletal collapse

The induction of disulfidptosis by natural products requires specific metabolic contexts characterized by elevated SLC7A11 expression and concurrent glucose deprivation. The polycyclic polyprenylated acylphloroglucinol gaudichaudione H exploits the metabolic paradox of system X^c-^ by inducing non-canonical, autophagy-mediated NRF2 activation, which drives the pathological overexpression of SLC7A11 (85). Under conditions of glucose starvation, this elevated cystine importation exhausts the intracellular NADPH pool, as NADPH is depleted during the reduction of cystine to cysteine (85). The resulting unreduced intracellular disulfides induce aberrant intermolecular disulfide cross-linking among actin cytoskeleton proteins, specifically targeting the reactive Cys374 residue of actin filaments to trigger catastrophic cytoskeletal infrastructure collapse and lytic death (85). Furthermore, natural flavonoids such as apigenin function as competitive inhibitors of the glucose transporter GLUT1, downregulating glucose uptake across multiple tumor models (86).

Because raw phytochemicals frequently suffer from poor systemic bioavailability, cutting-edge pharmacological strategies now integrate such metabolic interceptors into advanced nanodelivery platforms. For instance, when apigenin is encapsulated within multimodal bio-nanostructures, this engineered and sustained inhibition of glucose entry rigidly restricts NADPH regeneration. This synchronously drives disulfidptosis through cystine-mediated actin cross-linking, cuproptosis through the impairment of GSH-mediated copper chelation, and ferroptosis through GPX4 inactivation, achieving a triple regulated cell death phenotype and demonstrating the transformative potential of nanomedicine in NP delivery (87).

It is imperative to note that NP-induced alternative RCDs rarely operate in isolation. Instead, they exhibit extensive molecular cross-talk, frequently converging on shared upstream stress hubs, such as severe ROS bursts and mitochondrial dysfunction. For instance, excessive lipid peroxidation triggered by NPs not only dictates ferroptotic cascades but can simultaneously facilitate the oxidative cleavage of gasdermins, thereby initiating concurrent pyroptosis. Elucidating this intricate RCD interactome is crucial for rationally designing multi-target NP regimens.

## 4 Synergistic therapeutic paradigms and resistance reversal

**Figure 2:**
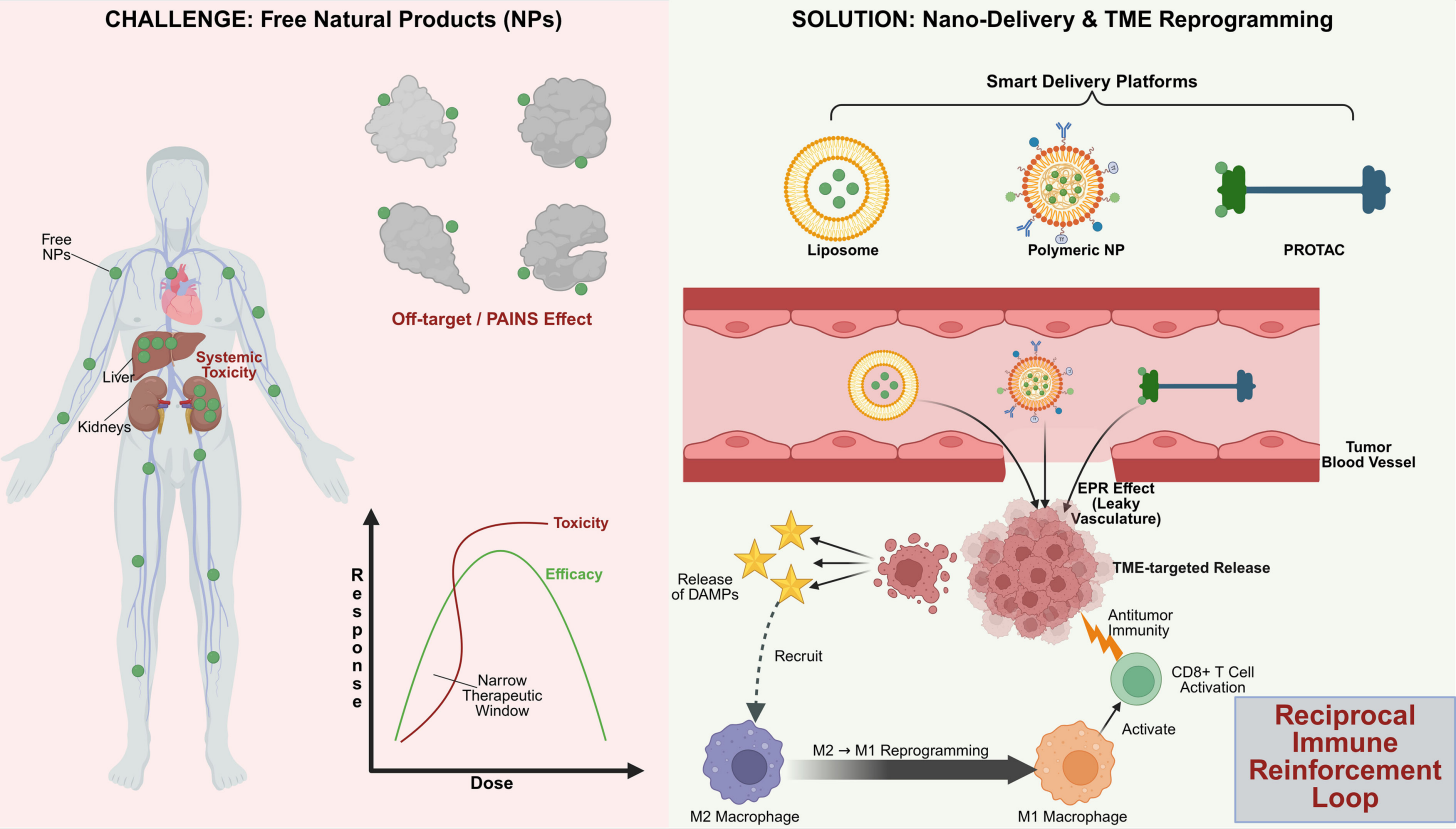
Synergistic therapeutic paradigms of NPs in overcoming MDR and remodeling the tumor microenvironment (created with BioRender.com). The schematic highlights three integrated preclinical strategies: (1) Synergistic Chemosensitization: NPs bypass canonical apoptosis resistance by shutting down pro-survival networks and initiating alternative RCD pathways, thereby resensitizing tumor cells to conventional chemotherapeutics (e.g., cisplatin, paclitaxel). (2) Reciprocal Reinforcement Loop: NP-induced RCD triggers immunogenic cell death via the release of DAMPs, promoting DC maturation and CD8+ T cell activation. Secreted IFN-γ from activated T cells further represses system X^c-^ (SLC7A11/SLC3A2), amplifying the initial ferroptotic and disulfidptotic stress. (3) Smart Nano-co-delivery Systems: Advanced nanocarriers, such as biomimetic vesicles and cascade nanoreactors, ensure the spatiotemporal synchronized release of NPs and targeted agents within the TME to optimize pharmacokinetics and maximize the therapeutic index.

NPs possess clinical translation potential beyond their roles as monotherapeutic cytotoxic agents; they function as multi-tiered network modulators that dismantle MDR and remodel the immunosuppressive tumor microenvironment (TME). By integrating front-line conventional therapies with phytochemical-mediated non-apoptotic mechanisms and advanced nanodelivery engineering, current combination strategies have established three distinct preclinical paradigms (**Figure 2**).

### 4.1 Synergistic chemosensitization: bypassing apoptosis resistance blockades

The failure of conventional chemotherapy and targeted therapies is frequently driven by defects in the canonical apoptotic machinery or ABC transporter-mediated drug efflux. As chemosensitizers, NPs (e.g., curcumin, resveratrol, and shikonin) suppress pro-survival signaling networks (such as NF-κB, PI3K/AKT/mTOR) while simultaneously initiating alternative non-apoptotic death pathways—including ferroptosis, necroptosis, and pyroptosis—thereby bypassing defective apoptotic cascades in drug-resistant tumors (88).

Reversal of Efflux and Metabolic Redirection: Curcumin functions as a broad-spectrum chemosensitizer across multiple drug-resistant models. Transcriptomic analyses indicate that in irinotecan-resistant colorectal cancer, curcumin reduces the half-maximal inhibitory concentration (IC_50_) by suppressing ABC transporters and UGTs (89). Curcumin also redirects apoptosis-resistant cells toward alternative death modalities; in multidrug-resistant lung cancer cells (e.g., doxorubicin-resistant H69AR), it synergistically sensitizes cells to doxorubicin by inducing ferroptosis-associated oxidative stress, leading to intracellular iron overload and lethal lipid peroxidation (90).

#### 4.1.1 Disrupting compensatory antioxidant responses

Platinum-based chemotherapeutics (e.g., cisplatin) induce DNA damage and concurrently trigger compensatory antioxidant stress responses in tumor cells. Natural products exploit these redox vulnerabilities to reverse resistance. In cisplatin-resistant ovarian cancer, shikonin overcomes resistance by upregulating HMOX1, which promotes Fe^2+^ accumulation, generates ROS, and downregulates GPX4 to trigger ferroptosis (91). In gastric cancer models, the combination of DHA and cisplatin demonstrates mechanistic complementarity, as DHA directly inhibits GPX4 to induce ferroptosis, resulting in a synergistic cytotoxic effect (92).

### 4.2 Reciprocal reinforcement with immunotherapy: the RCD-immunity feedback loop

The efficacy of immune checkpoint blockade (e.g., anti-PD-1/anti-PD-L1) depends on the density of T cell infiltration within the TME. Non-apoptotic RCD induced by NPs triggers ICD, providing a stimulus to reverse immune tolerance. This interaction operates as a bidirectional reciprocal reinforcement loop (93).

#### 4.2.1 ICD induction and T−cell recruitment

Cell lysis resulting from NP-induced ferroptosis or pyroptosis releases DAMPs, including calreticulin translocation to the plasma membrane and extracellular secretion of HMGB1 and ATP. In head and neck squamous cell carcinoma, an Fe^3+^-shikonin nanomedicine amplified Fenton-reaction-driven lipid peroxidation and GPX4 suppression. The resulting ferroptotic damage promoted DC maturation and increased infiltration of CD8+ cytotoxic T lymphocytes in the spleen and tumor stroma (93).

#### 4.2.2 IFN−γ−mediated feedback regulation

Activated CD8^+^ T cells secrete IFN-γ into the TME. IFN-γ transcriptionally represses SLC3A2 and SLC7A11 (system X^c−^), impairing cystine uptake and amplifying baseline ferroptotic stress (94). IFN-γ also stimulates ACSL4 to alter tumor cell lipid composition, increasing arachidonic acid incorporation into membrane phospholipids and rendering tumor cells susceptible to lipid peroxidation (95). This bidirectional cycle counteracts tumor immune evasion mechanisms.

### 4.3 Nanomedicine-driven co-delivery platforms: reshaping spatiotemporal pharmacokinetics

Although natural products exhibit synergistic potential *in vitro*, their *in vivo* translation is limited by pharmacokinetic barriers and disparities in distribution volume relative to chemotherapeutic agents. To ensure that combined agents reach tumor cells at predefined ratios, nano-co-delivery systems have emerged as a necessary pharmacological strategy. Several platform designs have been developed to address these challenges.

#### 4.3.1 Carrier−free prodrug nanoassemblies for coordinated release

To overcome the low drug-loading capacity of conventional nanocarriers, carrier-free prodrug nanoassemblies link agents via TME-cleavable bonds. For example, paclitaxel and DHA can be conjugated through a thioether bond to form self-assembled nanoparticles. After cellular uptake, elevated intracellular ROS cleaves the thioether linkage, releasing both agents in a coordinated manner. DHA amplifies ROS via the Fenton reaction and inactivates GPX4 to induce ferroptosis, while PTX further augments ROS, achieving synergistic ferroptotic-chemotherapy (96).

#### 4.3.2 Multimodal biomimetic vesicles and disulfidptosis induction

Advanced nanoplatforms integrate metabolic interception to trigger novel RCDs. Bilayer self-assembly engineered vesicles have been developed to disrupt intracellular glutathione, intentionally inducing disulfidptosis-enhanced cuproptosis for tumor immunotherapy (97). Disulfidptosis nanoinducers (such as CYBC NPs) co-delivering cystine and the GLUT1 inhibitor BAY-876 selectively trigger disulfidptosis-mediated cytoskeletal collapse in SLC7A11-high triple-negative breast cancer. This metabolic disruption generates ICD and CTL activation, inhibiting tumor recurrence (98).

#### 4.3.3 Toxicity attenuation and synergistic efficacy

An advantage of multi-drug nanomedicine is toxicity mitigation. In the co-delivery of celastrol and curcumin via multifunctional nanomedicine, the self-assembling platform achieves synergistic anti-hepatocellular carcinoma efficacy while curcumin’s hepatoprotective properties counteract celastrol-induced hepatotoxicity, achieving the dual endpoints of enhanced efficacy and reduced systemic toxicity (99).

## 5 Limitations and future perspectives

Despite extensive preclinical evidence supporting the efficacy of NPs in modulating non-apoptotic RCD, their clinical translation faces significant bottlenecks. Overcoming these barriers requires a paradigm shift from empirical application toward precision pharmacology, advanced structural engineering, and mechanistically driven clinical trial design. The primary translational bottlenecks and corresponding precision oncology strategies for NP-mediated RCD modulation are outlined in **Table 2**.

**Table 2:** Translational bottlenecks and precision medicine strategies for NP-mediated RCD modulation.

Translational domain	Current bottlenecks & limitations	Proposed precision oncology/advanced technology strategies	Key biomarkers & examples	Ref.
Patient Stratification & Diagnostics	Unstratified patient selection leads to inflated preclinical efficacy and clinical trial failures; lack of baseline molecular profiling.	Establish pre-treatment stratification systems; deploy liquid biopsies (ctDNA, tumor-derived exosomes) for dynamic real-time tracking of RCD sensitivity.	Ferroptosis: ACSL4/GPX4 expression ratio. Pyroptosis: GSDME promoter methylation. Cuproptosis: FDX1/LDH levels. Disulfidptosis: SLC7A11 high/GLUT1 inhibition.	(45, 101–104)
Medicinal Chemistry & Pharmacokinetics	Target ambiguity, unpredictable off-target toxicities (PAINS), poor aqueous solubility, and short systemic half-lives.	Utilize AI-driven lead optimization (e.g., G2D-Diff) for ADMET improvement; integrate PROTAC technology to convert NPs into specific target degraders.	Example: HIM-PROTAC or HyT utilizing NP scaffolds for ubiquitin-proteasome degradation of GPX4 at nanomolar doses.	(106–109)
Adaptive Drug Resistance	Tumors undergo epigenetic and metabolic plasticity to evade induced non-apoptotic RCDs, establishing novel compensatory networks.	Systematically monitor emerging orthogonal resistance pathways via CRISPR screens; implement multi-target combinations to disperse evolutionary pressure.	Emerging Resistance Nodes: FSP1-CoQ10 axis, SCD1-mediated MUFA enrichment, ATP7A/B copper efflux, mTORC2-GSDME signaling.	(111–115)
Nanomedicine Delivery & Synergy	Disparate distribution volumes and clearance rates between chemotherapeutics and NPs prevent optimal synergistic ratios *in vivo*.	Design TME-responsive (pH/GSH/ROS) smart nano-co-delivery systems and metal-free cascade nanoreactors to ensure spatiotemporal consistency.	Example: Bilayer self-assembly engineered vesicles for synchronized disulfidptosis-cuproptosis; GLUT1-inhibitor nanoplatforms.	(19, 97–99)
Clinical Trial Endpoints	Historical trials lack mechanistically driven designs and rely on non-specific readouts (e.g., general MDA levels) failing to verify target engagement.	Incorporate pathway-specific *in vivo* PD endpoints to validate targeted NP intervention mechanisms accurately.	Specific PD Endpoints: Hyperoxidized PRDX3 (Ferroptosis), DLAT oligomerization (Cuproptosis), specific GSDME cleavage fragments.	(41, 117–120)

### 5.1 Lack of precision patient stratification and dynamic biomarkers

Current preclinical and clinical studies largely administer NPs without pre-screening patients based on tumor molecular profiles. This unstratified strategy often leads to overestimated preclinical efficacy and subsequent clinical trial failures. To address this translational gap, stratification systems based on pre-treatment baseline molecular signatures are urgently needed (100). Histologically, the expression ratio of ACSL4 to GPX4 has been validated as an independent predictor of ferroptosis sensitivity (101); GSDME promoter hypermethylation serves as a key indicator for evaluating pyroptotic potential (102); and FDX1 abundance combined with plasma lactate dehydrogenase (LDH) levels provides a stratification basis for cuproptosis induction (103). Susceptibility to disulfidptosis is highly dependent on the metabolic vulnerability created by SLC7A11 overexpression combined with GLUT1 inhibition (45). For dynamic monitoring, liquid biopsies assessing circulating lipid peroxidation products (e.g., MDA and 4-HNE), analyzing ferroptosis-related genes (FRGs) in tumor-derived exosomes, and utilizing circulating tumor DNA (ctDNA) to track the evolution of RCD-related mutations may provide non-invasive tools to adjust personalized therapeutic strategies in real time (104). Moving forward, the empirical blanket administration of NPs must be replaced by rigorous patient stratification. Clinical success ultimately depends on implementing these biomarker-guided protocols to identify patient cohorts most likely to respond to specific RCD-inducing interventions.

### 5.2 Target ambiguity and future breakthroughs in medicinal chemistry

Although the multi-target polypharmacology of NPs helps overcome single-target resistance, it also introduces target ambiguity and unpredictable off-target toxicities. For instance, the biphasic dose-response (hormesis) of certain polyphenols can paradoxically induce systemic inflammation or hepatotoxicity and act as pan-assay interference compounds (PAINS). Furthermore, pharmacokinetic deficiencies, including poor aqueous solubility, extensive first-pass metabolism, and extremely short systemic half-lives, severely limit their bioavailability. While advanced nanomedicine platforms theoretically resolve these PK issues, they introduce complex Chemistry, Manufacturing, and Controls (CMC) challenges. The scale-up of sophisticated nanoreactors is frequently hampered by batch-to-batch inconsistency, high production costs, and a lack of standardized regulatory guidelines (e.g., FDA, EMA). Resolving these barriers requires integration with advanced medicinal chemistry (105). AI-driven lead optimization (e.g., using graph neural networks and the generative model G2D-Diff) can systematically optimize ADMET profiles while preserving the bioactivity of natural scaffolds (106). A more advanced application is proteolysis targeting chimera (PROTAC) technology. Certain NPs, such as oridonin and piperlongumine, have been shown to recruit MDM2 and KEAP1, respectively, expanding the ligand library for E3 ligases (107); these naturally derived E3 ligands provide additional tools for constructing PROTAC molecules targeting key resistance proteins. NP-based GPX4-targeted degraders (e.g., HIM-PROTAC and the hydrophobic tag degrader HyT) can achieve ubiquitin-proteasome degradation of GPX4 at nanomolar concentrations, overcoming the poor selectivity of traditional covalent inhibitors (108). Integration of TME-responsive (e.g., ROS, pH, or GSH-sensitive) prodrug designs and nano-co-delivery systems may further enhance the bioavailability and tumor-targeted accumulation of NPs (109).

### 5.3 Insufficient understanding of emerging resistance to non-apoptotic RCDs

Tumor cells exhibit substantial metabolic and epigenetic plasticity. When NPs are used to block apoptosis and activate alternative RCDs, malignant cells can undergo adaptive reprogramming that leads to acquired resistance (110). Recent genome-wide CRISPR-Cas9 screens have revealed multi-layered defense networks in tumors. For ferroptosis, alongside the classical GPX4 axis, tumors have evolved parallel antioxidant systems, including the FSP1-CoQ10 axis and DHODH (111). SCD1 upregulation driven by the PI3K/AKT/mTOR pathway promotes enrichment of monounsaturated fatty acids (MUFAs) in cell membranes, remodeling the lipid profile to resist peroxidation; gain-of-function mutations in MBOAT1/2 and NRF2 also contribute to ferroptosis evasion (112, 113). For pyroptosis, tumors not only silence GSDME through epigenetic mechanisms but also utilize mTORC2-mediated phosphorylation to promote ubiquitination and degradation of the GSDME N-terminus (114). For cuproptosis and disulfidptosis, major resistance pathways include ATP7A/B-mediated copper efflux, metabolic shifts toward glycolysis, and maintenance of NADPH homeostasis via IDH1 and ME1 (115). Moving forward, systematic application of genome-wide CRISPR-Cas9 screens in longitudinal preclinical models will be essential to delineate the full landscape of resistance alleles and inform rational combination strategies that disperse evolutionary selective pressure across multiple orthogonal pathways. Future preclinical studies should systematically monitor these emerging resistance mechanisms and use rational combinations with targeted therapies or immunotherapies to disperse the evolutionary selective pressure across multiple orthogonal pathways.

### 5.4 Limitations of clinical trials and pharmacodynamic endpoint design

Despite abundant data from *in vitro* and animal models, purified NPs that have advanced to Phase II/III clinical trials remain extremely scarce. The current landscape of representative clinical trials investigating phytochemicals in combination with conventional therapies is summarized in **Table 3**. Traditional clinical trials often lack mechanistically driven designs and biomarker-guided patient stratification (116). For example, curcumin exhibits PAINS properties, which lead to false-positive results *in vitro* and a biphasic dose-response that promotes proliferation at clinically achievable blood concentrations, contributing to a standstill in its clinical translation (117). Although artemisinin derivatives such as DHA demonstrate favorable pharmacokinetics and ferroptosis-inducing potential, relevant trials have not incorporated appropriate endpoints (118). Accelerating clinical translation requires optimization of pharmacodynamic (PD) endpoint designs. Conventional MDA or 4-HNE assays lack RCD specificity and cannot accurately reflect *in vivo* target engagement (119). Future clinical designs should incorporate highly specific biochemical markers, such as monoclonal antibodies against hyperoxidized PRDX3 to assess ferroptosis (120), or quantification of DLAT oligomerization to confirm cuproptosis (41), thereby validating the *in vivo* targeted intervention effects of NPs at the mechanistic level. These findings and proposed strategies inform the conclusions summarized in Section 6.

**Table 3:** Summary of representative clinical trials investigating natural products in combination with conventional therapies for cancer.

Natural product	Combination therapy	Cancer type	Trial phase & status	ClinicalTrials.gov ID	Key outcomes/observations
Curcumin	Paclitaxel	Advanced Breast Cancer	Phase II (Completed)	NCT03072992	Demonstrated high ORR and excellent tolerability; reduced paclitaxel-induced systemic inflammation.
Curcumin	FOLFOX (5-FU/Oxaliplatin)	Colorectal Cancer	Phase IIa (Completed)	NCT01490996	CUFOX trial: Safe and tolerable; showed potential synergistic efficacy in improving median PFS.
Artesunate	Standard Chemotherapy	Advanced Solid Tumors	Phase I/II (Completed)	NCT02353026	Well-tolerated with no dose-limiting toxicities; promising disease control rate mediated by enhanced oxidative stress.
Resveratrol	Bortezomib	Multiple Myeloma	Phase II (Completed)	NCT00920803	Co-administration encountered unacceptable nephrotoxicity in specific cohorts, highlighting the need for careful dose monitoring.
EGCG	Radiotherapy + Chemotherapy	Stage III Breast Cancer	Phase II (Active)	NCT02596682	Evaluated for reversing treatment resistance and mitigating radiation-induced dermatitis through redox modulation.
Genistein	Gemcitabine + Erlotinib	Pancreatic Cancer	Phase II (Completed)	NCT00508638	Modest survival benefit but established a manageable safety profile for combining flavonoids with frontline TKIs/chemo.

**Figure 3:**
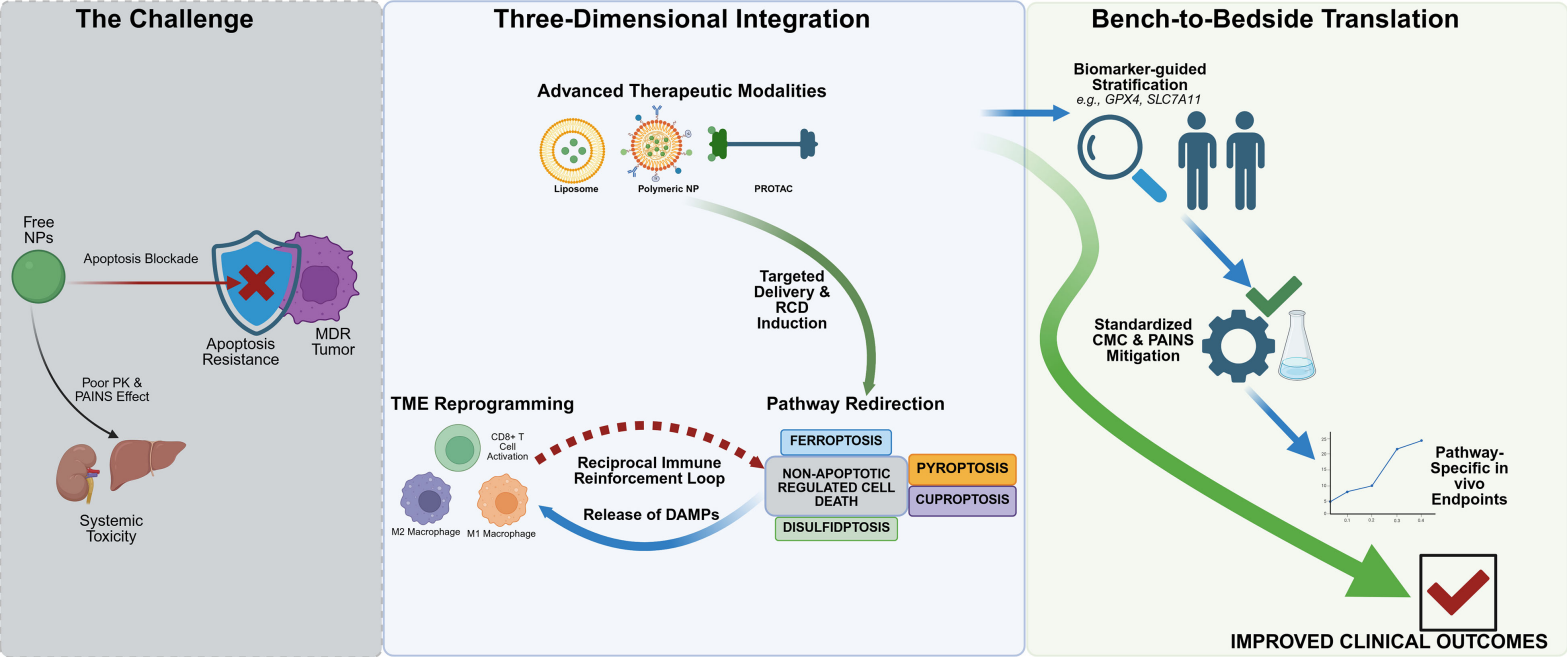
A translational roadmap for NP-mediated non-apoptotic RCD in cancer therapy (created with BioRender.com). (Left) Free NPs face severe preclinical bottlenecks, including apoptosis-resistant MDR and systemic/PAINS toxicity. (Middle) A three-dimensional therapeutic engine overcomes these barriers by utilizing smart nanomedicines/PROTACs for targeted delivery. This induces non-apoptotic RCDs and releases DAMPs, which subsequently reprogram the TME via a reciprocal immune reinforcement loop. (Right) Successful clinical translation hinges on three critical pillars: biomarker-guided patient stratification, standardized CMC manufacturing, and pathway-specific pharmacodynamic endpoints, ultimately driving improved clinical outcomes.

The core logic and translational roadmap of this review—from NP-mediated RCD induction to MDR reversal and clinical application—are conceptually summarized in **Figure 3**.

## 6 Conclusion

The era of relying solely on canonical apoptosis-inducing agents for cancer therapy is increasingly challenged by acquired MDR. This review highlights the profound practical significance of utilizing NPs to deliberately orchestrate alternative RCD pathways—specifically ferroptosis, pyroptosis, cuproptosis, and disulfidptosis. By bypassing classical apoptotic blockades and triggering immunogenic cell death, NPs offer a highly viable, actionable pharmacological strategy to overcome refractory MDR and resensitize the immunosuppressive tumor microenvironment.

However, the ultimate value of these mechanistic insights strictly hinges on their clinical translation. Moving from preclinical observations to clinical reality requires overcoming critical bottlenecks, most notably the ‘PAINS’ properties, systemic off-target toxicities, and poor bioavailability of phytochemicals. Therefore, future research must forcefully pivot away from conventional empirical *in vitro* screening toward advanced translational engineering and rigorous safety evaluation.

To achieve definitive clinical success, three translational pillars are indispensable: (1) integrating TME-responsive nanomedicines and AI-driven PROTAC technologies to ensure spatiotemporal precision and optimize ADMET profiles; (2) implementing rigorous, biomarker-guided patient stratification (e.g., assessing baseline GPX4, SLC7A11, and FDX1 levels) to identify responsive cohorts prior to intervention; and (3) establishing pathway-specific *in vivo* pharmacodynamic endpoints for future clinical trials. By bridging the gap between natural product polypharmacology and precision oncology frameworks, this review provides a definitive roadmap for translating non-apoptotic RCD modulators from the bench to the bedside.
